# Elevated Levels of IL-10 and G-CSF Associated with Asymptomatic Malaria in Pregnant Women

**DOI:** 10.1155/2010/317430

**Published:** 2010-07-12

**Authors:** Nana O. Wilson, Tameka Bythwood, Wesley Solomon, Pauline Jolly, Nelly Yatich, Yi Jiang, Faisal Shuaib, Andrew A. Adjei, Winston Anderson, Jonathan K. Stiles

**Affiliations:** ^1^Department of Microbiology, Biochemistry & Immunology, Morehouse School of Medicine, Atlanta, GA 30310, USA; ^2^Department of Epidemiology, School of Public Health, University of Alabama at Birmingham (UAB), Birmingham, AL 35294-0022, USA; ^3^Department of Pathology, University of Ghana Medical School, Korle-Bu, Accra, Ghana; ^4^Department of Biology, Howard University, Washington, DC 20059, USA

## Abstract

In sub-Saharan Africa, approximately 30 million pregnant women are at risk of contracting malaria annually. Nearly 36% of healthy pregnant women receiving routine antenatal care tested positive for *Plasmodium falciparum* HRP-II antigen in Ghana. We tested the hypothesis that asymptomatic HRP II positive pregnant women expressed a unique Th1 and Th2 phenotype that differs from healthy controls. Plasma from healthy (*n* = 15) and asymptomatic (*n* = 25) pregnant women were evaluated for 27 biomarkers (IL-1b, IL-1ra, IL-2, IL-4, IL-5, IL-6, IL-7, IL-8, IL-9, IL-10, IL-12, IL-13, IL-15, IL- 17, Eotaxin, bFGF-2, G-CSF, GM-CSF, IFN-*γ*, IP-10, MCP-1, MIP-1*α*, MIP-1*β*, PDGF-bb, RANTES, TNF, and VEGF) associated with Th1 and Th2 cytokine homeostasis. IL-10 and G-CSF levels were elevated in the asymptomatic group when compared with the healthy group (*P* = .031 and .041, resp.). The median ratios of IL-1*β*:5, IL-1*β*:10, IL-1*β*:G-CSF, IL-1*β*:Eotaxin, IL-12:G-CSF, IL-15:10, IL-17:G-CSF, IL-17:Eotaxin, TNF:IL-4, TNF:IL-5, and TNF:G-CSF were significantly different among the two groups. Thus, asymptomatic malaria carriage may be linked to circulating levels of IL-10 and G-CSF.

## 1. Introduction

The World Health Organization estimates that around 250 million cases of malaria infections and 1–3 million associated deaths globally are reported annually [1]. Infection with *Plasmodium falciparum* has a wide spectrum of manifestations that are classified into three main clinical groups: asymptomatic (presence of malaria parasite without malaria symptoms), mild, and severe malaria. In malaria-endemic areas, a significant proportion of individuals considered asymptomatic, harbor parasites without presenting signs of clinical malaria [2]. The significance of such asymptomatic infections in the broader context of malaria transmission has been evaluated in diverse situations using complementary approaches [3–6]. Variant-specific immunity has been used to explain the low-grade infection during extended periods without clinical symptoms [7]. Studies suggest that long-term asymptomatic carriage may represent a form of tolerance to parasites in individuals such that asymptomatic carriage may protect these individuals from developing severe malaria [3–5]. Older individuals are more likely to develop uncomplicated malaria or asymptomatic parasitemia [8]. In high malaria endemic areas, protection from severe malaria is acquired early during childhood, although it takes longer to be protected from less severe disease [9]. Although immunoprotective mechanisms clear a large proportion of infected erythrocytes, a subset persists in the circulation leading to asymptomatic parasitemia [4, 9]. 

Conversely, asymptomatic carriage may represent a mode of entry to symptomatic malaria, especially in young children [6, 10]. Many asymptomatic infections go undetected and untreated while causing little or no clinical manifestation. The extent of the prevalence of asymptomatic parasitemia in a given population is inversely related to the population's susceptibility to clinical disease [11, 12], but more importantly, asymptomatic individuals are major reservoirs of infection [13, 14]. Gouagna et al. [15] have demonstrated that parasites from asymptomatic individuals are more infectious to mosquitoes than are parasites from symptomatic individuals. These results have driven current presumptive intermittent antimalarial treatment strategy for asymptomatic individuals, regardless of their malaria infection status, to reduce malaria morbidity and mortality [16]. Furthermore, field surveys of malaria using microscopy have often failed to detect very low-level parasitemia, which is common among asymptomatic patients.

During successful pregnancies, maternal leukocytes produce predominately Th2-type cytokines to prevent initiation of inflammatory and cytolytic-responses that might damage the integrity of the placental barrier [17]. In response to invading pathogens, Th1-type cytokines may be produced that reverses the Th2-type bias [18]. The deviation in local and systemic immune response towards a Th2 bias may leave pregnant woman more open to Th1-dependent infections such as malaria thereby contributing in increased morbidity. A previous study in Ghana observed 36.3% of asymptomatic *P. falciparum* infection among pregnant women [19]. Similarly, Gabon and Cameroon have reported 57% and 82.4% asymptomatic infection prevalence respectively [20, 21] suggesting that pregnant women may present cytokine or chemokine levels that may confer less susceptibility to symptomatic malaria thereby harboring malaria parasite without showing any symptoms. The current study examined the immune modulators associated with asymptomatic parasitemia in pregnant women and compared with health-uninfected controls. The levels of malaria-associated cytokine, chemokine, and growth factors in asymptomatic healthy pregnant women were evaluated simultaneously to characterize and identify potential biological factors induced by asymptomatic *P. falciparum* infections that provide insights into malaria immunopathogenesis as well as targets for adjunctive therapies to prevent malaria morbidity and mortalities.

## 2. Methods

### 2.1. Study Subjects and Samples

Women who had a singleton and uncomplicated pregnancy with no clinical symptoms of malaria presenting at their third trimester for delivery at two hospitals in Kumasi Ghana, the Komfo Anokye Teaching Hospital and the Manhyia Polyclinic in Kumasi, November to December 2006 were enrolled after informed consent. Medical history before and during pregnancy was obtained and recorded. 746 women with ages ranging from 15 to 48 were enrolled with an average age of 26.8 years. Venous blood samples were collected after enrollment. Red blood cells and plasma were separated and stored at −80°C until ready to use. Hemoglobin levels, malaria antigens in the blood as well as presence of helminthes in stool samples were determined. Malaria antigen levels were determined by using a Malaria Antigen ELISA kit (CELISA, Cellabs, Australia). This kit measures HRP II production during growth and multiplication [22] of *P. falciparum* at a specificity of 96% and sensitivity of 98% in whole blood or plasma and can detect *P. falciparum* parasites at a limit of detection of 0.001% [23]. Study participants were categorized into asymptomatic carriers (ACs) and healthy controls (HCs). The AC group were positive for HRPII while HC were negative for HRPII. Determination of hookworms, *Ascaris lumbricoides*, *Strongyloides stercoralis,* and *Trichuris trichura* was done as previously described [19]. Stool samples were processed within 12 hours of collection and examined microscopically within 1 hour of preparation to avoid missing hookworm ova [19].

### 2.2. Multiplexed Microsphere Cytokine Immunoassay

Forty plasma samples were randomly selected from 746 samples from pregnant women (15 healthy control and 25 asymptomatic carriers) and were evaluated simultaneously for circulating levels of 17 cytokines (IL-1*β*, IL-1ra, IL-2, IL-4, IL-5, IL-6, IL-7, IL-9, IL-10, IL-12, IL-13, IL-15, IL-17, G-CSF, GM-CSF, TNF, IFN-*γ*), 7 chemokines (IL-8, Eotaxin, IP-10, MCP-1, MIP-1*α*, MIP-1*β*, RANTES), and 3 growth factors (bFGF-2, PDGF-bb, VEGF) using a commercially available multiplex bead-based cytokine assay coupled with the Luminex system (Austin, TX) and human-specific magnetic beads Bio-Plex Cytokine Kit (Bio-Rad, Hercules, CA). Biological factor levels were measured using optimal concentrations of standards and antibodies according to the manufacturer's instructions. Briefly, plasma samples were diluted 1 : 4 in Bio-Plex sample diluents and an eight point, broad range cytokine standard was prepared by serial dilution from a cytokine stock. Fifty microliters of diluted sample or standard were transferred to 96-well filtration plate containing anticonjugated beads. The plate was shaken for 30 seconds at 1,100 rpm, reduced to 300 rpm, and incubated for 60 minutes at room temperature. After incubation and washes, detection antibody (2 mg/mL) was added and incubated for 30 minutes. After 3 washes, streptavidin-PE was added and incubated for 10 minutes. Beads were mixed before reading on the Bio-Plex Suspension Array System. The data was analyzed using Bio-Plex Manager software with 5-parameter logistic (5PL) regression curves.

### 2.3. Statistical Analysis

To normalize the distribution of cytokine values, the data was log transformed. Differences in individual biological factor concentrations between HC and AC groups were compared using nonparametric Kruskal-Wallis one-way analysis of variance (ANOVA) on ranks. Dunn's method was used for all pairwise multiple comparison procedures to isolate the group or groups that differ from the others. The differences in proinflammatory or angiostatic to anti-inflammatory or angiogenic cytokine median ratios were compared between the two groups using ANOVA to determine the balance or disequilibrium of Thl and Th2 determinants between the groups. Box plots of median biomarker concentrations with 25th and 75th percentiles were plotted. Values outside the bars for 10th and 90th percentiles were plotted as points. Statistical significance for each biological factor was set at a two-tailed *P* < .05. SigmaPlot software version 10 (Systat Software Inc., San Jose, CA) was used to calculate statistics and plot graphs.

## 3. Results

### 3.1. Patient Characteristics

Nearly one-third (30.2%) of the 746 participants were primigravidae and 98.7% had at least one prenatal visit prior to delivery with most occurring in the third trimester of pregnancy [19]. However, among the 40 random samples selected for the present study, the participants were at least secundigravidae. The prevalence of *P. falciparum* among the participants was 36% and 16.6% had malarial and helminth coinfection [19]. Out of the 40 randomly selected samples, 13% HC and 44% AC were anemic (hemoglobin < 11 g/dL) (Table 1). The mean hemoglobin levels were significantly higher in the HC (12.63 ± 1.17) than AC (10.98 ± 2.32), *P* = .017. None of the HC had helminth infection and 16% of the AC samples had one or multiple helminth infection. The helminth infection did not appear to affect the anemic outcome observed in the AC group (Table 1).

### 3.2. Biomarker Levels in Healthy Controls and Asymptomatic Carriers

**Figure 1:**
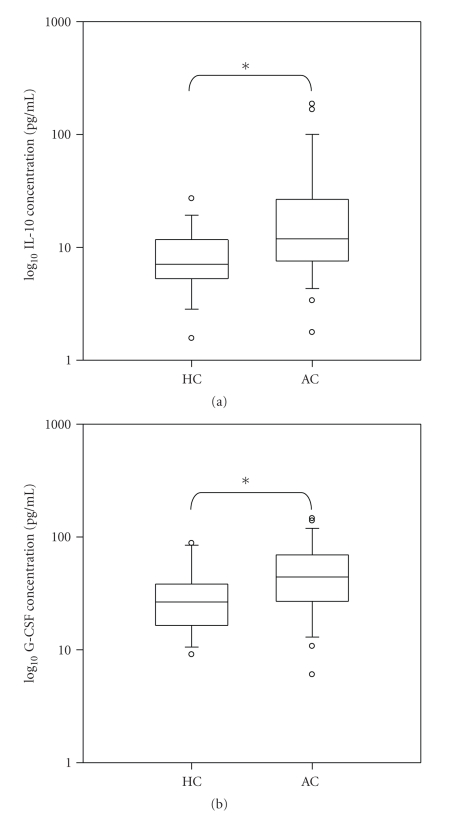
Concentration of plasma IL-10 and G-CSF in pregnant women with asymptomatic malaria (*n* = 25) and healthy controls (*n* = 15). Box plots represent median log concentration (pg/ml) and 25th and 75th percentiles. Outliers are shown as points outside the 10th and 90th percentile bars. HC represents healthy controls. AC denotes asymptomatic carriers. *P < .05 is statistically significant.

Pairwise comparisons were used to determine the levels of significance of the differences between the plasma biological factor levels of the two groups (HC and AC) after controlling for age and primigravidae. The plasma levels of 27 biological factors were grouped into proinflammatory, anti-inflammatory, and angiogenic/angiostatic biomarkers and analyzed. The proinflammatory and angiogenic/angiostatic levels demonstrated marginal changes but did not show statistically significant differences between the HC and AC groups (data not shown). Among the anti-inflammatory biomarkers evaluated, IL-10 and G-CSF were significantly (*P* < .05) elevated among the AC group compared to HC group (Figure 1). In addition, among the AC group comparing helminth infection with absence of helminth infection, there were no significant differences in the peripheral biological factor levels suggesting that helminth infection did not influence the peripheral biological factor levels.

### 3.3. Biomarker Ratios in Healthy Controls and Asymptomatic Carriers

To determine the influence of parasite antigen on Th1and Th2 homeostasis, plasma proinflammatory (IL-1*β*, IL-2, IL-6, IL-8, IL-12, IL-15, IL-17, GM-CSF, IFN-*γ*, MCP-1, MIP-1*α*, MIP-1*β*, RANTES, TNF) or angiostatic (IP-10, IL-12) to anti-inflammatory (IL-1ra, IL-4, IL-5, IL-6, IL-9, IL-10, IL-13, G-CSF, Eotaxin) or angiogenic (IL-7, IL-8, bFGF-2, PDGF-bb, VEGF) cytokine median ratios were compared between the two groups (HC and AC). Comparing the median ratios of proinflammatory to anti-inflammatory factors and angiogenic to angiostatic biomarkers among HC and AC groups, biomarker median ratios (IL-1*β*:5, IL-1*β*:10, IL-1*β*:G-CSF, IL-1*β*:Eotaxin, IL-12:G-CSF, IL-15:10, IL-17:G-CSF, IL-17:Eotaxin, TNF-*α*:IL-4, TNF:IL-5, TNF-*α*:G-CSF) were statistically different among HC and AC (Table 2).

## 4. Discussion

Asymptomatic malaria (presence of malaria parasite without symptoms) is prevalent in highly endemic areas in Africa [24]. It is suggested that asymptomatic parasitemia confers partial immunity and protection against symptomatic malaria [5, 25]. On the other hand, asymptomatic individuals constitute a remarkably large reservoir for malaria transmission or could be a precursor in the progression of symptomatic form of malaria. This study was conducted after reviewing data generated in a previous study [19] and finding a remarkably high number (36%) of asymptomatic patients recorded as healthy controls. We acknowledge that the cross sectional measurement of parasitemia and biological factor levels in host blood and the lack of follow-ups to determine the true impact of asymptomatic parasitemia is a limitation of this study. Nevertheless, the importance of our observation is predicated on recent studies that indicate that individuals with asymptomatic parasitemia may have a higher risk of developing symptomatic malaria than those without evidence of parasitemia [26]. In our study, the AC group had lower hemoglobin level compared to the HC group (Table 1). This lower level of hemoglobin could be due to the presence of chronically low parasitemia causing extensive destruction of red blood cells. Many asymptomatic infections are undetected by microscopy and thus most asymptomatic individual do not receive required treatment thereby rendering them susceptible to the risks associated with both symptomatic and asymptomatic malaria. This persistent problem has resulted in the proposed presumptive intermittent treatment strategy for asymptomatic individuals, regardless of their malaria infection status, to reduce malaria morbidity and mortality [16]. This therapeutic method may adversely result in accelerating the development of resistance against anti-malarial drugs suggesting an urgent need to determine the true infection status before treatment. This approach is useful in decreasing the prevalence of asymptomatic malaria and the negative consequence of drug resistance to anti-malarial drugs.

The present study examined a broad range of immune mediators, including cytokines, chemokines, and markers of apoptosis and angiogenesis in healthy and asymptomatic malaria carrier pregnant women. The immunological relevance of these biological factors associated with asymptomatic malaria have been previously established [27, 28]. Due to the lack of concurrent studies of inflammatory, apoptotic, and angiogenic factors in appropriate time-matched symptomatic patients, the results are discussed in context. Proinflammatory Th1-type cytokines are thought to be critical to the control of exoerythrocytic and erythrocytic *P. falciparum *infection [29, 30], but their exaggerated production may also contribute to organ damage, particularly in the brain in cerebral malaria cases. It is widely accepted that anti-inflammatory Th2-type cytokines downregulate Th1-derived cytokines. Th2-type cytokines, such as IL-10, regulate Th1-cytokines and prevent severe forms of malaria in some animal models [31]. The regulation of TNF levels by IL-10 appears to contribute to the prevention of severe malarial anemia in humans [32–34]. However, the role that IL-10 plays may depend on its levels, since very high levels of IL-10 have been associated with severe malaria in humans [35] and some animal models [36]. In our analysis, higher levels of IL-10 were observed in the AC group than the HC group. IL-10 is a key cytokine involved in both protection and immunopathology during malaria. High levels of IL-10 may be beneficial by reducing the inflammatory response, but may be detrimental by decreasing antiparasitic cellular immune responses. IL-10 is an anti-inflammatory cytokine that acts in part by blocking monocyte and macrophage production of inflammatory cytokines such as IL-6, TNF, and IL-l [37]. Animal studies have suggested that IL-10 may play a regulatory role that modulates susceptibility to parasite infection. In particular, IL-10 inhibits microbicidal activity of IFN-*γ*-treated macrophages against intracellular parasites such as *Toxoplasma gondii* [38], *Trypanosoma cruzi* [39] and *Leishmania major* [40], and the killing of extracellular *Schistosoma mansoni schistosomulas* [41]. IL-10 has been previously observed to be elevated during malarial episodes in nonpregnant [42, 43] and pregnant women [44]. Both increased and decreased levels of IL-10 have been associated with poor malaria outcomes. Low levels of IL-10 or low IL-10 to TNF ratios were associated with severe malarial anemia in African children [32, 34], while high IL-10 levels were associated with reduced ability to eliminate malaria parasitemia in Tanzanian children [45]. Thus, the high levels of IL-10 observed in the AC group may reduce their ability to eliminate or clear malaria parasitemia as seen in the HC.

Cytokines may stimulate beneficial immunological responses in falciparum malaria by inducing acute phase responses, inhibition of parasitic growth, and clearance of vascular parasites and debris. Neutrophils are stimulated by cytokines such as TNF or INF-*γ* in order to increase parasite destruction [46]. In the present study, it seems that G-CSF may be involved in controlling *P. falciparum *parasitemia, which ultimately results in the asymptomatic condition observed in our AC group. G-CSF has both anti-infective and anti-inflammatory effects [47]. G-CSF enhances phagocytosis and increases chemotaxis and antibody-dependent cytotoxicity suggesting its involvement in anti-malarial defense by activating neutrophils and acting as an immunomodulatory factor [47]. Surprisingly, G-CSF levels were elevated in the AC compared to HC suggesting that this factor may be involved in maintaining low parasitemia levels observed in the asymptomatic individual. Functional studies on the association of G-CSF and IL-10 with asymptomatic malaria are much needed to reveal their contribution to the spread and severity of malaria. 

Maternal leukocytes produce predominately Th2-type cytokines shifting the cytokine balance towards Th2-type response during pregnancy to prevent initiation of inflammatory that might damage the integrity of the placental barrier [17]. In response to invading pathogens,Th1-type cytokines may be produced to reverse the Th2-type bias [18] to aid in elimination of parasites by enhancing phagocytic activity of macrophages, generating reactive oxygen intermediates, and stimulating the proliferation of T cells [48]. However, our median ratio result indicates that there was significance bias towards a Th2-type profile in the AC group with low pro- to anti-inflammatory ratios compared to the HC group (Table 2). This deviation in immune response towards a Th2 bias may explain the asymptomatic observation in the AC group due to the fact that this group has a less robust Th1-type response to eliminate malaria parasites compared to the HC group. In addition, the Th2 bias may leave pregnant AC more open to Th1-dependent infections that may result in increased morbidity in pregnancy.

We acknowledge that this study has limitations that can be investigated in future studies. The current study did not include cytokine profiles of nonpregnant women and pregnant women with malaria as well as asymptomatic nonpregnant women to determine whether our observations were unique to pregnant women. However, IL-10 has been observed to be elevated during malarial episodes in nonpregnant [42, 43] and pregnant women [44]. Although our study lacked malaria positive pregnant women, comparing the results to other studies in which malaria positive pregnant women were included suggests that the levels of peripheral IL-10 in the AC group were lower than pregnant women with placental malaria [49]. In addition, studies in murine models of malaria have suggested that IL-10 response during infection may be associated with the disease exacerbation [36].

To the best of our knowledge, there are no comprehensive prospective studies that describe cytokine profile during pregnancy, therefore, we do not know whether the observation made in this study may have been influenced by pregnancy-specific conditions. However, the increase in asymptomatic cases [19–21, 50, 51] highlights the alarming potential of malaria as a serious public health issue underscoring the need for tighter surveillance [50]. Asymptomatic individuals not only serve as a source of maintenance of intense malaria transmission during the peak season but could also perpetuate resistance through the inappropriate use of drugs [51]. 

In summary, cytokine, chemokine, and growth factor evaluation in asymptomatic healthy pregnant women reveal an association between increased plasma concentrations of IL-10 and G-CSF. Thus, susceptibility to symptomatic malaria in pregnancy may be associated with high levels of IL-10 and G-CSF and dysregulation of pro and anti-inflammatory factors in pregnant women. In the absence of studies explaining the functional implication of this finding for the asymptomatic condition observed in these pregnant women, we propose that assessment of these biological factors in conjunction with rapid detection of *P. falciparum* HRP-II and PCR-based methods could be a useful tool for predicting risk for asymptomatic malaria carriage.

## Figures and Tables

**Table 1:** Description of the pregnant women in the study. HC denotes healthy control and AC denotes asymptomatic carrier. The values for hemoglobin was *m* *e* *a* *n* ± *S* *D*.

Characteristics	HC	AC	*P*-value
No. of Subjects	15	25	
HRP II/Malaria	Negative	Positive	
Mean Hemoglobin Level (g/dL)	12.63 ± 1.17	10.98 ± 2.32	.017
% Anemia (Hb < 11 g/dL)	13	44	
Ascaris Infection	0	2	
Hookworm Infection	0	1	
Trichiuris Infection	0	1	
Strongyloides Infection	0	0	
Enterobius Infection	0	0	
Taenia Infection	0	0	

**Table 2:** Comparison of selected plasma biomarker median ratios between the HC and AC Group. Statistical comparison of median ratios of proinflammatory, angiostatic versus anti-inflammatory and angiogenic factors in different subject groups. *P* < .05 considered statistically significant.

Proinflammatory or Angiostatic versus Anti-inflammatory or Angiogenic Biomarker Median Ratio	AC	HC	Overall *P* Value
IL-1*β*:IL-5	2.73	3.31	.021
IL-1*β*:IL-10	0.74	1.06	.040
IL-1*β*:G-CSF	0.21	0.33	.003
IL-1*β*:Eotaxin	0.18	0.31	.001
IL-12:G-CSF	0.261	0.420	.040
IL-15:IL-10	0.163	0.848	.039
IL-17:G-CSF	1.102	1.723	.036
IL-17:Eotaxin	1.004	1.627	.030
TNF:IL-4	15.335	24.518	.047
TNF:IL-5	9.974	15.510	.031
TNF:G-CSF	0.831	1.855	.002
